# A combined inelastic neutron scattering and simulation study of the ^3^He@C_60_ endofullerene†

**DOI:** 10.1039/d3cp02253f

**Published:** 2023-06-08

**Authors:** Mohamed Aouane, Jeff Armstrong, Mark Walkey, Gabriela Hoffman, George R. Bacanu, Richard J. Whitby, Malcolm H. Levitt, Stéphane Rols

**Affiliations:** a Institut Laue-Langevin, BP 156 Grenoble 38042 France rols@ill.fr; b ISIS Facility, Rutherford Appleton Laboratory, Harwell Oxford Didcot Oxfordshire OX11 0QX UK; c School of Chemistry, University of Southampton Southampton SO17 1BJ UK

## Abstract

The ^3^He@C_60_ endofullerene consists of a single ^3^He atom entrapped inside a C_60_ fullerene cage. The confining potential, arising from the non-covalent interaction between the enclosed He atom and the C atoms of the cage, is investigated by inelastic neutron scattering. These measurements allow us to obtain information in both energy (*ω*) and momentum (*Q*) transfers in the form of the dynamical structure factor *S* (*Q*, *ω*). Simulations of the *S* (*Q*, *ω*) maps are performed for a spherical anharmonic oscillator model. Good agreement between the experimental and simulated data sets is achieved.

## Abstract 2

Uniting inelastic neutron scattering and numerical simulations to fully understand the dynamics of ^3^He atoms confined within C_60_ cages.

## Graphical abstract

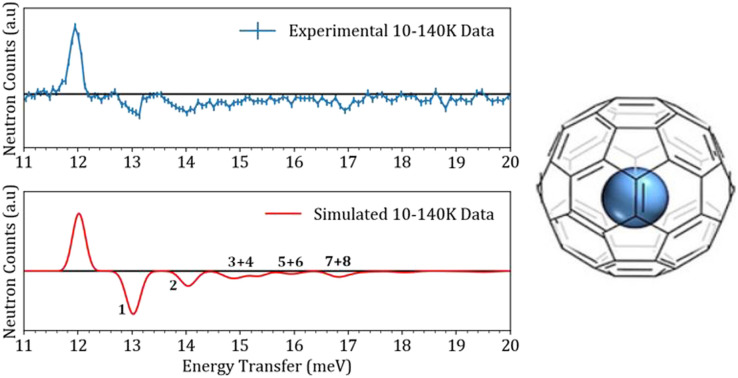

## 1 Introduction

The study of confined particles is at the heart of any quantum physics textbook, including examples of particles moving in a harmonic oscillator potential well and the particle trapped in a box.^1^ Studying these examples constitutes a starting block to deepen our understanding of the quantum dynamics of more realistic systems. Endofullerene molecules, A@C_60_, consist of small atomic or molecular entities trapped inside a highly symmetric C_60_ cage.^2^ The molecular surgery^3,4^ method, consisting of opening an orifice in the fullerene cage allowing the introduction of light molecules and atoms, has paved the way for the synthesis of multiple confined quantum rotors such as H_2_@C_60_,^5^ H_2_O@C_60_,^6^ HF@C_60_,^7^ HD@C_60_^8^ and CH_4_@C_60_.^9^ At cryogenic temperatures, quantum effects play a central role in the dynamics of the entrapped species, with the rotational and translational motions becoming quantized. These phenomena have been probed by a wide range of spectroscopic techniques,^10^ including inelastic neutron scattering (INS),^7,11–18^ infrared spectroscopy^7,15,19–21^ and NMR.^22–24^ Alongside these experimental investigations, numerical calculations have been used to compute the INS spectrum for various endofullerenes.^25–29^ These calculations were crucial to understand the features observed experimentally, showing the necessity of performing simulations to understand neutron scattering spectra.^30^

Noble gas endofullerenes^3,8,31^ represent the simplest molecular arrangement in the endofullerene family. They consist of a single atom moving while confined within a C_60_ cage and experiencing a van der Waals interaction with the carbon atoms. Recent breakthroughs in the synthesis method of endofullerenes have made it possible to obtain the helium endofullerenes ^3^He@C_60_ and ^4^He@C_60_ with high purity, high filling factor, and in a sufficiently large quantity for neutron scattering investigations.^4,8^

The translational modes of both the ^3^He and ^4^He atoms were recently investigated by INS and terahertz spectroscopy,^18^ with the experimental results interpreted using an anharmonic oscillator potential to describe the potential energy surface (PES). The potential parameters were then determined by fitting the experimental data. The experimental PES was then compared to those derived from quantum chemistry calculations and from empirical potentials with parameters^32^ found in the literature.

This paper focuses on further testing of the anharmonic oscillator potential describing the interaction between a He atom and the C_60_ cage. This is done by comparing experimental INS data, gathered as a function of both momentum transfer *Q* and energy transfer *ω*, to simulations obtained using the anharmonic oscillator^18^ potential energy surface description.

The paper is divided into two parts: the first part where new INS results obtained through direct and indirect geometry time of flight spectrometers at different temperatures will be discussed. In the second part, these new experimental results will be compared to simulations performed using the anharmonic^18^ potential.

## 2 Sample preparation and experimental setup

### 2.1 Sample preparation

A 1084 mg ^3^He@C_60_ sample (filling factor of 45%) alongside a 196 mg ^4^He@C_60_ sample (filling factor of 35%), both in powder form, were used to conduct the INS measurements. Both samples were synthesized by the “molecular surgery” method, with a novel solid-state reaction at the closing stage of the cage.^8^ As an extra precaution, trace impurities of H_2_O@C_60_ were removed from both samples by recirculating high-performance liquid chromatography (HPLC) on Cosmosil Buckyprep columns.^8^ This was done to avoid any parasitic signal from hydrogen which would be much stronger than the one coming from He isotopes due to their low neutron scattering cross sections and the very high hydrogen neutron scattering cross section.

### 2.2 Experimental details

Experimental data was gathered at both the Institut Laue-Langevin (ILL) and the ISIS Neutron and Muon Source large scale facilities.

PANTHER,^33^ is a direct geometry time of flight (ToF) spectrometer using thermal neutrons located in the reactor hall of the ILL, and allows the probe of different parts of the (*Q*, *ω*) space depending on the incident energy (*E*_i_) chosen and gives *S*(*Q*, *ω*) maps as a result of covering the [5°, 135°] angular range.

**Fig. 1:**
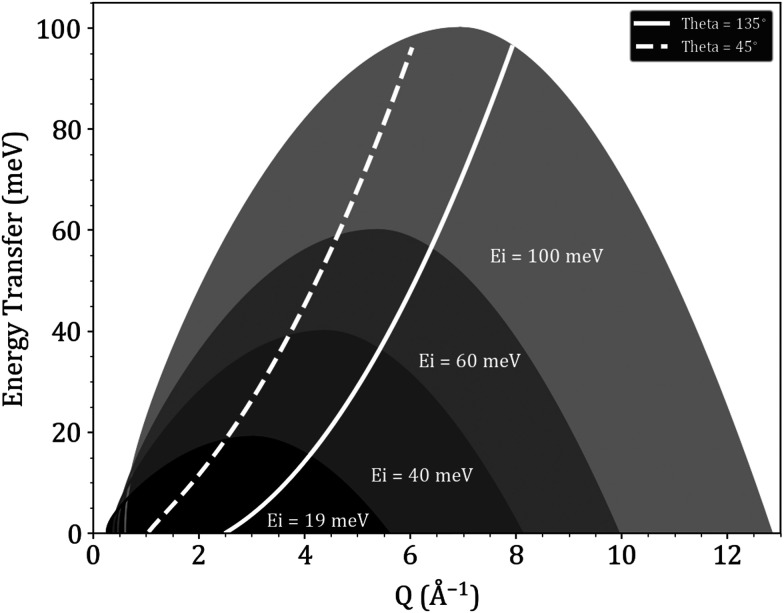
Domains in the (*Q*, *ω*) space probed by PANTHER at different incident energies (*E*_i_) and by the two TOSCA detector banks at 45° and 135°. For visualisation only energies up to 100 meV, in the positive energy transfer region, were plotted.

TOSCA^34^ on the other hand, is an indirect geometry vibrational spectrometer at ISIS, where the incident neutron beam is polychromatic (white beam) and the final neutron energy is fixed to the 3.8 meV value. Unlike PANTHER, TOSCA offers information as a function of energy transfer only, and has two detector banks, one at 45° and one at 135°. ToF and vibrational spectrometers are complementary to each other, as ToF spectrometers offer a global vision of the (*Q*, *ω*) space, whereas the vibrational spectrometers offer the possibility of focusing on a specific part of said domain with a better resolution. Fig. 1 shows the different regions in (*Q*, *ω*) space that are probed by both PANTHER and TOSCA, and how both are complementary to each other.

All neutron data sets in this paper were reduced through the Mantid^35^ software.

## 3 Experimental results

### 3.1 PANTHER results

This paper focuses on the ^3^He isotope as it yields the main results. This choice is dictated by the low sample mass of ^4^He@C_60_ and the comparatively weaker neutron scattering cross section of ^4^He compared to ^3^He. The experiments on PANTHER and TOSCA were performed using the ^3^He@C_60_ sample described in the previous section. The experiments were conducted at a temperature of 1.6 K with the sample loaded into Al foil to reduce the contribution of the usually used sample cans in the data sets.

**Fig. 2:**
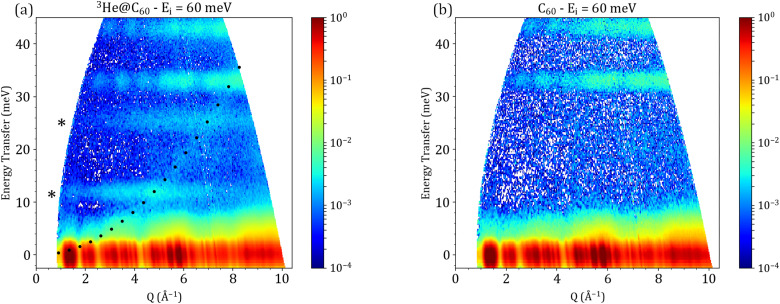
Experimental dynamical structure factor *S*(*Q*, *ω*) for ^3^He@C_60_ (a) and mass matching blank C_60_ (b) measured on PANTHER at *E*_i_ = 60 meV at 1.6 K. The features marked with stars correspond to the first two translational modes of the entrapped ^3^He atom. The dispersive feature present in the ^3^He@C_60_ PANTHER measurement at *E*_i_ = 60 meV in figure panel (a) can be perfectly reproduced by the recoil curve (black dots) of ^4^He gas that was left over in the PANTHER cryostat.

Fig. 2 shows *S*(*Q*, *ω*) maps measured on PANTHER at an incident energy *E*_i_ = 60 meV for both ^3^He@C_60_ and a mass matching blank C_60_. The characteristic gap of C_60_ spanning the 8 to 33 meV region,^36,37^ shows the presence of two features in the ^3^He@C_60_ measurement, which correspond to the translational motions of the entrapped ^3^He atom. At low temperatures, the translational motion of the entrapped ^3^He atom becomes quantized.

The position in energy of these two modes is around 12 and 24 meV, which matches well with the previously published results.^18^ The mode at 12 meV corresponds to the fundamental translational mode *n* = 0 to 1 and has a maximum in *Q* which is around 4.6 Å^−1^.

The momentum transfer (*Q*) information obtained from direct geometry ToF spectrometers can provide additional confirmation that the second observed feature at around 24 meV corresponds to a transition from *n* = 0 to 2. Using the formalism developed by Parker *et al.*^38^ which can be summarized as 
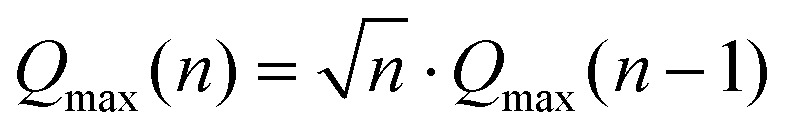
, we can confirm that the second mode is indeed an *n* = 0 to 2 transition. 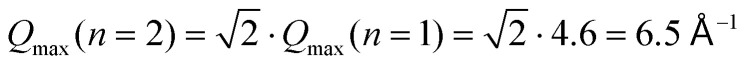. The value given by the formula above corresponds to the maximum in *Q* for the second observed mode at 24 meV. In a harmonic oscillator picture the *n* = 3 mode would be around 36 meV with a maximum in *Q* at around 11.3 Å^−1^. Unfortunately, the fact that intense C_60_ modes appear from 33 meV onward, coupled with the fact that the region in (*Q*, *ω*) space probed at *E*_i_ = 60 meV on PANTHER does not reach this *Q* range, hampers the observation of the *n* = 0 to 3 transition.

Focusing on the first two observed translational modes of ^3^He, a better resolution measurement on PANTHER was performed by using a lower incident energy. The resulting *S*(*Q*, *ω*) map for *E*_i_ = 40 meV on PANTHER is shown in panel (a) of Fig. 5.

The better resolution measurement on PANTHER further confirms the previous INS measurements on IN1-LAGRANGE,^18^ as the *n* = 2 level is split into two sublevels, one for each of the possible *l* values of 0 and 2.

The measurements provide data sets that are a function of both energy and momentum transfers, which will serve as a reference for testing our anharmonic oscillator potential energy surface.

### 3.2 TOSCA results

The second set of experiments shown and discussed in this paper were performed on TOSCA at three different temperatures: 10, 70 and 140 K. The higher temperature runs populate higher (*n*, *l*) states, allowing the observation of “hot bands” corresponding to transitions coming from states (*n*, *l*) ≠ (0, 0). The same ^3^He@C_60_ sample was used again, this time loaded into an Al sample can specific to the TOSCA instrument. The lack of a blank C_60_ measurement was influenced by both time constraints and by the blue-shift effect observed in C_60_ modes due to the presence of a ^3^He atom inside the cage, as discussed in our previous study.^18^

**Fig. 3:**
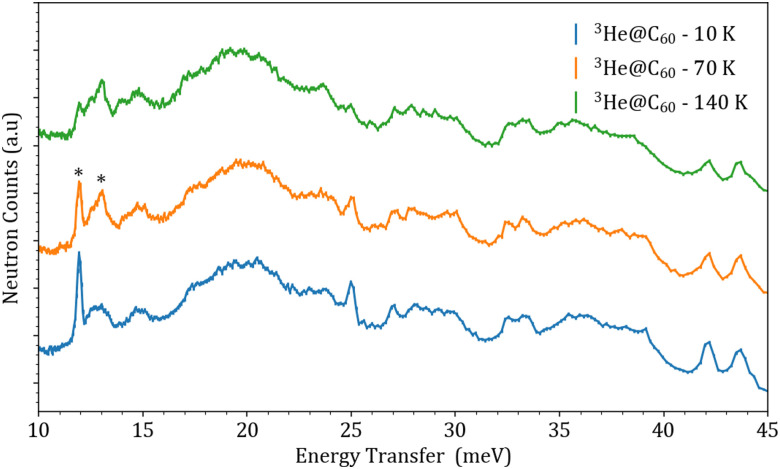
The TOSCA measurements for ^3^He@C_60_ at all three temperatures for the 135° detector bank. The INS spectra were shifted along the *y*-axis for visualisation, with some of the “hot bands” marked with asterisks.

Fig. 3 shows the resulting measurement at all three temperatures for the 135° detector bank.

**Fig. 4:**
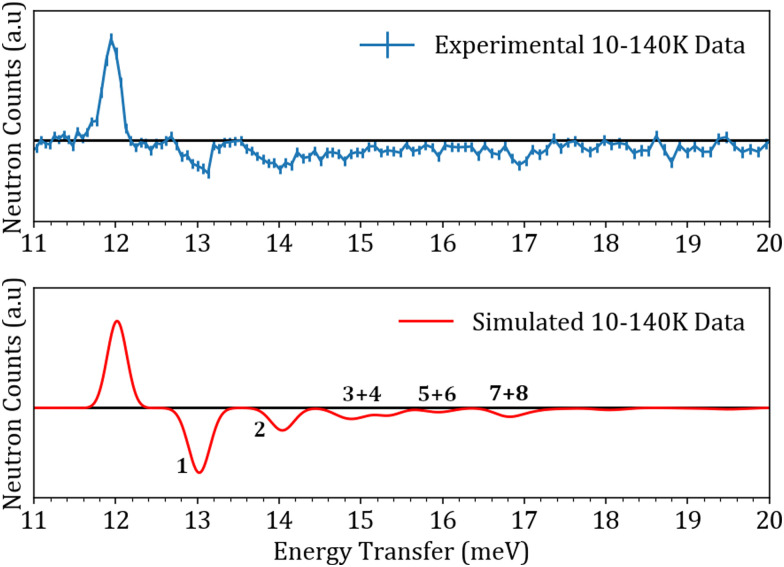
Comparison between the experimental and simulated sets of data corresponding to the 10–140 K subtraction. The peaks labelled by numbers correspond to the “hot bands”.

The features marked with a star in the TOSCA measurements correspond to ‘hot bands’, which are transitions that arise from excited energy levels (*n*, *l*). The hot bands are observed in the 10 to 20 meV energy range, alongside a drastic decrease in intensity of the fundamental peak at 12 meV with increasing temperature. The broad feature covering the 15 to 25 meV range corresponds to phonon modes coming from the aluminium sample can. In order to remove the large Al sample can signal and clean up the INS spectrum, the higher temperature run at 140 K was subtracted from the 10 K run. This results in the appearance of “positive” and “negative” peaks which correspond to transitions coming from the (0, 0) ground state and from higher (*n*, *l*) states. The result of this subtraction is shown in Fig. 4.

## 4 Simulation results

### 4.1 INS simulations: theory

For this section, we propose the use of the anharmonic oscillator potential, a sum of *r*^2^, *r*^4^ and *r*^6^ terms, as described in eqn (1), with parameters shown in Table 1.

1 *V*(*r*) = *V*_2_*r*^2^ + *V*_4_*r*^4^ + *V*_6_*r*^6^

For this potential, the C_60_ cage is assumed as rigid with its icosahedral (*I*_h_) symmetry not playing an important role in this system, allowing us to approximate the C_60_ as a perfect sphere. This in turn, allows us to drop the angular dependency of the potential energy surface, leaving *r*, the distance from the centre of the cage, as the only variable: *V*(*r*, *θ*, *ϕ*) ≈ *V*(*r*). The PES can also be constructed using a superposition of sixty 6–12 Lennard-Jones type interactions using parameters from Pang and Brisse.^32^ This will be discussed in the supplementary information of this paper, where the sensitivity of this potential to the chosen C_60_ radius value will be shown and discussed.

**Table 1:** Best fit result for the three parameters describing the polynomial oscillator potential. Results published in our previous study^18^

Parameter	^3^He
*V*_2_ (meV Å^−2^)	25.80 ± 0.11
*V*_4_ (meV Å^−4^)	33.7 ± 0.6
*V*_6_ (meV Å^−6^)	27.86 ± 0.05

**Fig. 5:**
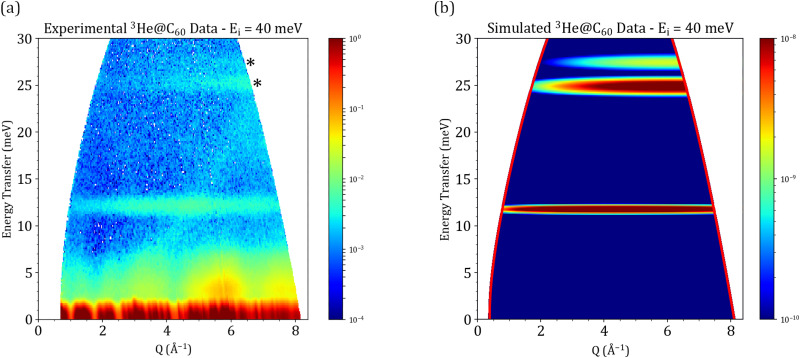
(a) Experimental data for ^3^He@C_60_ on PANTHER at *E*_i_ = 40 meV for a temperature of 1.6 K. (b) Simulation of the equivalent *S*(*Q*, *ω*) using the anharmonic oscillator PES. The simulation is in logarithmic scale to highlight the *n* = 2 split energy level marked by asterisks in panel (a).

Simulating the experimental INS data shown in Fig. 4 and 5 requires good knowledge of both the eigenvalues and eigenvectors of the entrapped ^3^He atom. In a neutron scattering experiment, the interaction process involves changes in both energy *ω* and momentum transfer *Q*. This can be summarized by the double differential cross section shown in eqn (2).^39,40^2
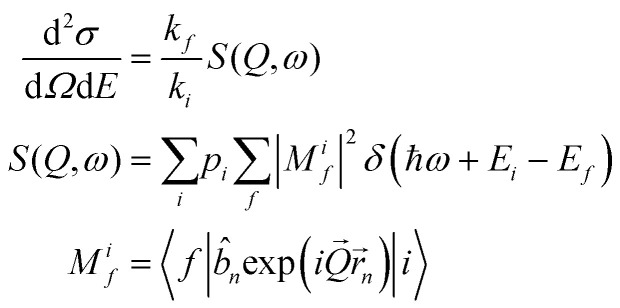
3
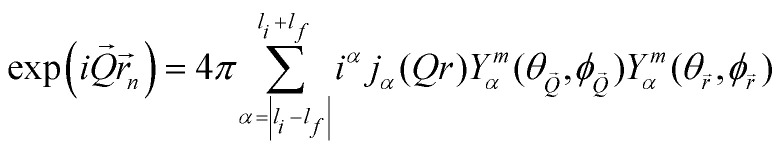
*M*^*i*^_*f*_ represents the scattering matrix element for a process involving a transition from an initial state |*i*〉 of energy *E*_i_ towards a final state |*f*〉 of energy *E*_*f*_ upon interaction with the incident neutron. *p*_*i*_ represents the population, derived through Boltzmann distribution, of the initial state |*i*〉 at a given temperature *T*. For the simulations of PANTHER data, the initial state |*i*〉 corresponds to the (*n*, *l*) = (0, 0) state. The calculation of the *M*^*i*^_*f*_ term is done in a completely numerical way through exploiting the development of the plane wave as a series^41^ as shown in eqn (3).

**Fig. 6:**
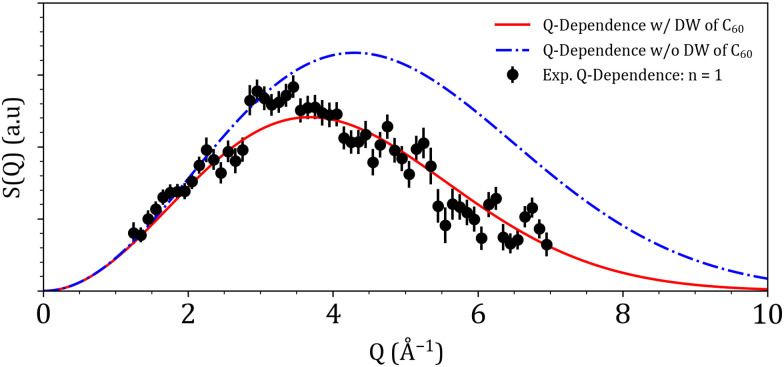
*Q*-dependence for the *n* = 0 to 1 transition of the entrapped ^3^He atom. Experimental data points from PANTHER are in black, the blue dash-dotted line and the red solid line represent the simulated *Q*-dependence without and with considering the Debye–Waller factor respectively.

As the ^3^He atoms are separated by a distance of at least 14 Å, these calculations assume that there is no interaction between two neighbouring ^3^He atoms, reducing the simulations to a one atom calculation problem. Despite the low temperature during the measurements, the C_60_ cages are still subject to thermal motions around their equilibrium position. In order for our simulations to take the lattice dynamics into account, the Debye–Waller factor written as exp{−Q^2^〈*u*^2^〉/3}, needs to be introduced. Accurate inclusion of the Debye–Waller factor in our INS simulations will prove essential for obtaining reliable results, as it allows for the correct modeling of the dynamical behavior of atoms in a crystal lattice at finite temperatures. This is shown in Fig. 6, where a simulation shows the effect of accurately taking into account the Debye–Waller factor. The simulation of the INS spectra was done in a fully numerical manner, where the radial Schrödinger equation was solved for different (*n*, *l*) states giving both the corresponding eigenvalue and eigenvector. Table 2 shows a comparison between the experimentally observed lines on PANTHER and the ones obtained numerically from the anharmonic oscillator potential energy surface. Table 2 also serves as a way to attribute the correct (*n*, *l*) quantum numbers to the three observed peaks on PANTHER at *E*_i_ = 40 meV at *T* = 1.6 K.

**Table 2:** Comparison between the experimental and simulated position in energy (in meV) of the transitions and their corresponding quantum transition for the anharmonic oscillator (A. O.) potential

PANTHER	A. O. PES	Transition
12.13 ± 0.02	11.72	(0, 0) to (1, 1)
25.31 ± 0.03	24.84	(0, 0) to (2, 2)
27.25 ± 0.08	27.42	(0, 0) to (2, 0)

### 4.2 INS simulations: PANTHER

Fig. 5 presents the simulation results of the PANTHER data sets, obtained by numerically evaluating the eigenvectors and eigenvalues of the entrapped ^3^He atom using the anharmonic oscillator PES. This data set was specifically chosen due to the clear splitting of the *n* = 2 level that could be observed in the experimental data. At a qualitative level, both the position in energy and the *Q*-dependence of the simulated features seem to agree well with the experimental data. In order to go more in-depth and seek a quantitative agreement, a cut along the *Q*-axis was performed for the *n* = 0 to 1 transition at 12 meV in both the experimental and simulated data sets. This transition was chosen due to the fact that experimentally, the full *Q*-dependence of the mode could be observed with a good resolution in the *E*_i_ = 40 meV PANTHER measurement. Fig. 6 shows the resulting cut along *Q* for the experimental and simulated data. Looking at the simulation, without the Debye–Waller factor of the host C_60_ cage taken into account, the general shape of the *Q*-dependence between the experimental and simulated data seems to be the same but with a poor agreement when it comes to the position of the maximum in *Q*. This discrepancy arises from an assumption made when performing the simulations. Earlier in the text, it was stated that the C_60_ cage is considered as rigid in space, implying that it was a fixed sphere in space with no dynamics. In reality, the C_60_ cages form a lattice that has its own dynamics even at temperatures as low as 1.6 K. In order to take the lattice dynamics into account, the mean square displacement, 〈*u*^2^〉, of the C_60_ lattice has to be taken into account to correct the simulated *Q*-dependence. This is done by taking the value determined by Copley *et al.*^42^ of 0.06 Å^2^, injecting it into the Debye–Waller factor exp {−*Q*^2^〈*u*^2^〉/3} then multiplying the simulated *Q*-dependence by it. Doing this results in a near-perfect agreement between the simulated and experimental *Q*-dependence for the first translational mode of ^3^He as shown in Fig. 6.

### 4.3 INS simulations: TOSCA

TOSCA was used to perform experiments at temperatures higher than 1.6 K. This meant that higher (*n*, *l*) states would be populated and allow the observation of hot bands as stated earlier. Table 3 shows the population of some of the (*n*, *l*) states through the Boltzmann population factor using the anharmonic oscillator potential. As mentioned in the experimental section, a subtraction of the 140 K data set from the 10 K set was performed in order to remove the contributions arising from the Al sample can. The result of this subtraction for both the experimental and simulated data sets is shown in Fig. 4.

**Table 3:** Percentage of population for different (*n*, *l*) states for the three measurement temperatures on TOSCA

*T* (K)	(0, 0) (%)	(1, 1) (%)	(2, 2) (%)	(2, 0) (%)	(3, 3) (%)
10	99.99	—	—	—	—
70	83.26	13.74	1.58	1.14	0.155
140	29.41	37.07	12.6	10.7	3.9

**Fig. 7:**
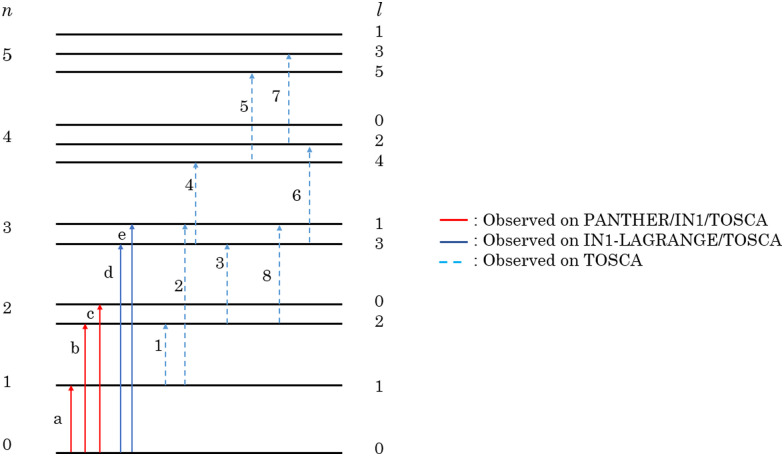
The energy diagram resulting from our three INS experiments on the entrapped ^3^He atom inside C_60_. Peaks labeled by letters were attributed in the previous study.^18^

The difference in widths between the experimental and simulated data sets can be attributed to the filling factor of the ^3^He endofullerene. If the filling factor is in the 40–70% range as is the case with this sample, the different encapsulated ^3^He atoms would see slightly different environments, leading to slightly different positions in energy for the same transition. This can explain why when looking at the experimental data, the simulations appear to be much sharper. These new INS experiments have allowed us to further test the anharmonic oscillator model, with parameters shown in Table 1, describing the He–C interaction. The qualitative and quantitative agreements between the experimental and simulated data sets has even allowed us to assign newly observed transitions, which would have been difficult to do if the model was an inaccurate description of the PES. The results from all of our INS experiments on TOSCA, PANTHER and IN1-LAGRANGE^18^ are shown in the form of an energy diagram in Fig. 7 showing the newly observed transitions with the corresponding quantum numbers.

## 5 Conclusions

This paper has shown the complementarity between direct geometry time of flight and vibrational neutron spectrometers and builds upon our previously released investigation.^18^ The use of PANTHER at different incident energies *E*_i_ at 1.6 K further confirmed the results from our previous study.^18^ More importantly, the use of PANTHER gives access to information in both energy transfer and momentum transfer *Q* which was not available for ^3^He@C_60_ until this study. The second set of experimental data was from TOSCA, and was performed at the higher temperatures of 10, 70 and 140 K in order to populate higher (*n*, *l*) states of the entrapped ^3^He atom. These sets of experimental data gave us benchmark tests for our simulations of INS data.

In order to simulate the INS data, we have adopted the previously published potential energy surface which consisted of a sum of *r*^2^, *r*^4^ and *r*^6^ terms. The validity of both the spherical approach and the parameters we have defined for the potential were then tested in different ways: the first one was to recreate the *S*(*Q*, *ω*) maps measured on PANTHER for ^3^He@C_60_ at *E*_i_ = 40 meV. The anharmonic oscillator PES gave a good qualitative agreement. A quantitative agreement was obtained through exploring the *Q*-dependence, a feature that can be obtained only through direct geometry time of flight neutron spectrometers. Once the C_60_ cage dynamics were taken into account through the Debye–Waller factor, a good quantitative agreement between the experimental and simulated data sets was obtained.

The second test for the anharmonic oscillator PES was the TOSCA *S*(*ω*) data. First, the population of the different (*n*, *l*) states was calculated through the Boltzmann population factor for three temperatures: 10, 70 and 140 K. A comparison between the experimental and simulated TOSCA data sets was performed. This comparison showed a good agreement between the two, which meant that the populations calculated by the anharmonic oscillator PES for the three temperatures provided an accurate description of the system. The fact that our experimentally derived potential passed both tests when simulating the data sets from different techniques confirms that our approach in describing the confining environment is a very valid one and has allowed us to observe and assign newly observed transitions.

## Author contributions

MW/GH/RJW: He@C_60_ synthesis. SR/MA: PANTHER experiments. JA/MA/SR: TOSCA experiments. MA/SR: INS simulations. MA/JA/GRB/RJW/MHL/SR: ILL and ISIS proposal writing. MHL/RJW/SR: securing funding through EPSRC and the ILL PhD program.

## Conflicts of interest

There are no conflicts to declare.

## Supplementary Material

CP-025-D3CP02253F-s001
