# Supplementary material for: A combined inelastic neutron scattering and simulation study of the 3He@C60 endofullerene
Source: Phys Chem Chem Phys. 2023 Jun 8;25(30):20295–301. doi: 10.1039/d3cp02253f (PMC10394999; doi:10.1039/d3cp02253f)
Supplement: CP-025-D3CP02253F-s001 [file CP-025-D3CP02253F-s001.pdf]

Electronic Supplementary Material (ESI) for Physical Chemistry Chemical Physics.
This journal is © the Owner Societies 2023

Supplementary Information: A Combined Inelastic Neutron Scattering and Simulation Study of the ^{3}He@C_{60} Endofullerene

Mohamed Aouane[‡1], Jeff Armstrong[2], Mark Walkey[3], Gabriela Hoffman[3], George R. Bacanu[3], Richard J. Whitby[3], Malcolm H. Levitt[3], and Stéphane Rols[1]

[1]Institut Laue-Langevin, BP 156, 38042 Grenoble, France
[2]ISIS Facility, Rutherford Appleton Laboratory, Harwell Oxford, Didcot, Oxfordshire, OX11 0QX, United Kingdom
[3]School of Chemistry, University of Southampton, Southampton, SO17 1BJ, United Kingdom
[‡]Current address: Same as affiliation 2.

1 ^{4}He@C_{60} INS Data:

As mentioned in the main text, measurements on both the ^{3}He and ^{4}He endofullerenes were performed. Figure 1 shows the resulting measurements on TOSCA for ^{4}He@C_{60} at 10 K. Due to the low sample mass and the low neutron scattering cross section of ^{4}He compared to ^{3}He, only the fundamental transition n = 0 to 1 could be observed for ^{4}He at around 9 meV.

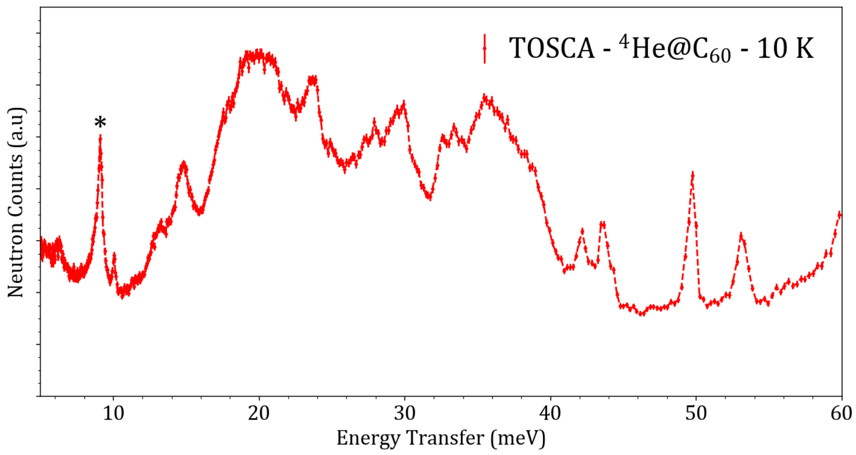

S. 1: The TOSCA measurement for ^{4}He@C_{60} at 10 K for the 135° detector bank. The feature marked with an asterisk corresponds to the fundamental translational mode of ^{4}He.

2 Lennard-Jones Potential Energy Surface:

As mentioned in the main text, another way of describing the PES of the entrapped He atom is to approximate the C_{60} as a sphere and consider the 6-12 Lennard-Jones potential (equation 1) with parameters shown in table 1.

$$v(r) = 4\varepsilon\left[\left(\frac{\sigma}{r}\right)^{12} - \left(\frac{\sigma}{r}\right)^{6}\right] \quad (1)$$

Interaction	σ (Å)	ε (meV)
He-C	2.971	1.61

Table 1: Parameters for the 6-12 Lennard-Jones potential describing the interaction between He and C, taken from Pang and Brisse[1].

Plugging the parameters in table 1 into equation 1 and integrating it over a sphere of radius R, we obtain equation 2

$$V(r) = \frac{15}{Rr}\left[\frac{A}{2}\left[(r+R)^{-4} - (r-R)^{-4}\right] - \frac{B}{5}\left[(r+R)^{-10} - (r-R)^{-10}\right]\right] \quad (2)$$

Equation 2 is the result of approximating the C_{60} as a perfect sphere, where A = $4\varepsilon\sigma^6$, B = $4\varepsilon\sigma^{12}$ with r representing the displacement of the He atom from the centre of the sphere and R the radius of the sphere. For this approximation, we consider R = 3.547±0.005 Å as determined from neutron diffraction[2].

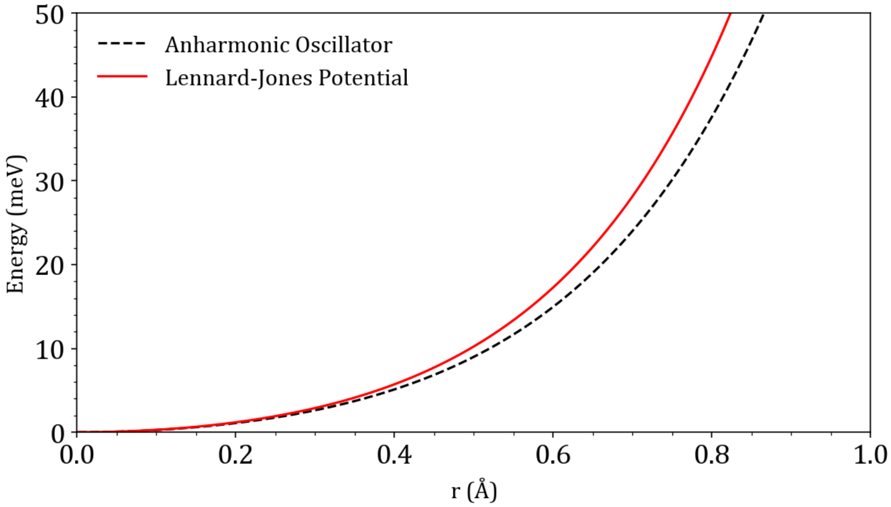

S. 2: Visual comparison between the anharmonic oscillator PES, with parameters shown in the main text (dashed black line), and the Lennard-Jones spherical approximation with parameters shown in table 1 (solid red line).

As a point of comparison, figure 2 shows a comparison between the anharmonic oscillator PES described in the main text and the Lennard-Jones PES in the spherical approximation. As can be seen, the differences between the two PES' become more apparent as energy increases.

PANTHER	LJ PES	A.O PES	Transition
12.13±0.02	12.46	11.72	$(0,0)$ to $(1,1)$
25.31±0.03	26.45	24.84	$(0,0)$ to $(2,2)$
27.25±0.08	29.28	27.42	$(0,0)$ to $(2,0)$

Table 2: Comparison between the experimental and simulated position in energy (in meV) of the transitions and their corresponding quantum transition for the anharmonic oscillator (A.O) and Lennard-Jones (LJ) potentials.

Table 2 shows a comparison of the experimentally features observed on PANTHER and the eigenvalues derived by both the anharmonic oscillator and Lennard-Jones potentials.

Figure 3 shows a comparison between the experimental data set measured on PANTHER for ^{3}He@C$_{60}$ and a simulated one using the Lennard-Jones PES showing the discreancy between the two data sets proving that the anharmonic oscillator discussed in the main text is a better description of the non-bonded interaction between the entrapped ^{3}He atom and the C$_{60}$ cage.

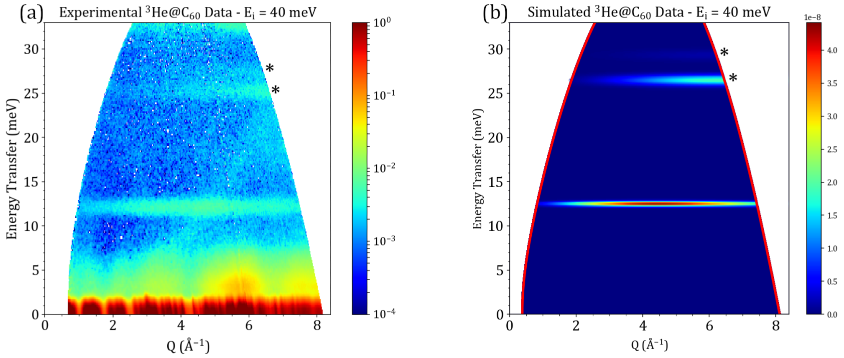

S. 3: Comparison between (a): the experimental PANTHER data for $^3He@C_{60}$ and (b): the simulated data using the Lennard-Jones PES shown in equation 2.

References

[1] L. Pang and F. Brisse, *The Journal of Physical Chemistry*, 1993, **97**, 8562–8563.

[2] F. Leclercq, P. Damay, M. Foukani, P. Chieux, M. Bellissent-Funel, A. Rassat and C. Fabre, *Physical Review B*, 1993, **48**, 2748.
